# The complete mitochondrial genome of brown algae: *Sargassum fusiforme* (Harvey) Setchell

**DOI:** 10.1080/23802359.2020.1715892

**Published:** 2020-01-24

**Authors:** Tao Liu, Yutong Cui, Xuli Jia, Binbin Chen, Zengling Ma, Huixi Zou, Shengqin Wang, Mingjiang Wu

**Affiliations:** a College of Life Sciences, Yantai University, Yantai, China;; b College of Life and Environmental Science, Wenzhou University, Wenzhou, China;; c College of Marine Life Sciences, Ocean University of China, Qingdao, China

**Keywords:** *Sargassum fusiforme*, Sargassaceae, phylogenetic analysis, mitochondrial genome

## Abstract

We describe the complete mitochondrial genome sequence of *Sargassum fusiforme*. This mitogenome is a circular molecule of 34,695 bp in length and had an overall GC content of 37.54%%. Gene annotation showed that 35 protein-coding genes, 2 open reading frames, 25 transfer RNA genes, and 3 ribosomal RNA genes. The phylogenetic tree based on Bayesian shows that *S. fusiforme* belongs to genus *Sargassum*, support current taxonomic systems.

*Sargassum fusiforme* (Harvey) Setchell, used to be regarded as a taxonomic synonym of *Hizikia fusiformis* (Harvey) Okamura (Kokubu et al. 2015), belongs to the Sargassaceae family of Fucales, Phaeophyta. It mainly grows along the rocky coastlines of China, Korea, and Japan (Yu et al. 2012; Hu et al. 2016). It is one of the most widely consumed seaweeds, not only as food, but also as traditional Chinese herbal medicine. This seaweed’s body is yellowish-brown in color, is succulent and is rich in nutrition. Besides, it is rich in polysaccharides, proteins, and microelements which are beneficial to human body (Ma et al. 2004; Xie et al. 2014; Samad et al. 2017). Due to the promising medical and food therapeutic effects, *S. fusiforme* have attracted a great deal of attention from both pharmaceutical and food industries.

Here, we report the complete mitochondrial genome of *S. fusiforme* and investigate the phylogenetic relationship with other related species based on mitochondrial proteins, to provide a genomic resource and to clarify phylogenetic relationship of this seaweed with other species in the Sargassaceae family. The specimen was collected from Wenzhou, Zhejiang Province, China (N27°50′25.19″, E121°01′23.36″), and stored at the Culture Collection of Seaweed at the Ocean University of China with an accession number 2015040102. Total DNA was extracted using the modified CTAB method (Doyle and Doyle 1990). Paired-end reads were sequenced using Illumina HiSeq × Ten system (Illumina, USA). De novo assembly and confirmation were conducted by SPAdes-3.10.1 (http://bioinf.spbau.ru/spades) (Bankevich et al. 2012). Sequence annotation was added using Geneious Prime. At last, the annotated sequence was submitted to GenBank with the accession number MN883537.

The complete mitochondrial genome of *S. fusiforme* was found to be 34,695 bp in length with 37.54% overall GC content. The nucleotide contents of A, T, G, and C in the mitochondrial genome were 26.70, 35.76, 21.60, and 15.94%, respectively. There are a total of 65 genes in the mitochondrial genome, comprising 35 protein-coding genes, 2 open reading frames, 25 transfer RNA genes and 3 ribosomal RNA genes. It is worth noting that all of Protein-encoding genes use ATG as a start codon, 25 protein-encoding genes used TAA as a stop codon and the remainings were TAG (8), TGA (4). All genes show the typical gene arrangement conforming to the Sargassaceae family consensus.

In order to better elucidate the phylogenetic relationship between *S. fusiforme* and its relative species, mitochondrial genome sequences downloaded from the GenBank database of 14 species were used to construct phylogenetic tree by the Bayesian method, with *Saccharina japonica* (GenBank accession number NC_013476) served as outgroup. Support values for each node were calculated from Bayesian posterior probability (BPP). The Phylogenetic tree (Figure 1) exhibited that *S. fusiforme* belongs to genus Sargassum, support current taxonomic systems. This is significant for further understanding of the phylogenetic evolution of the *S. fusiforme*.

**Figure 1.**
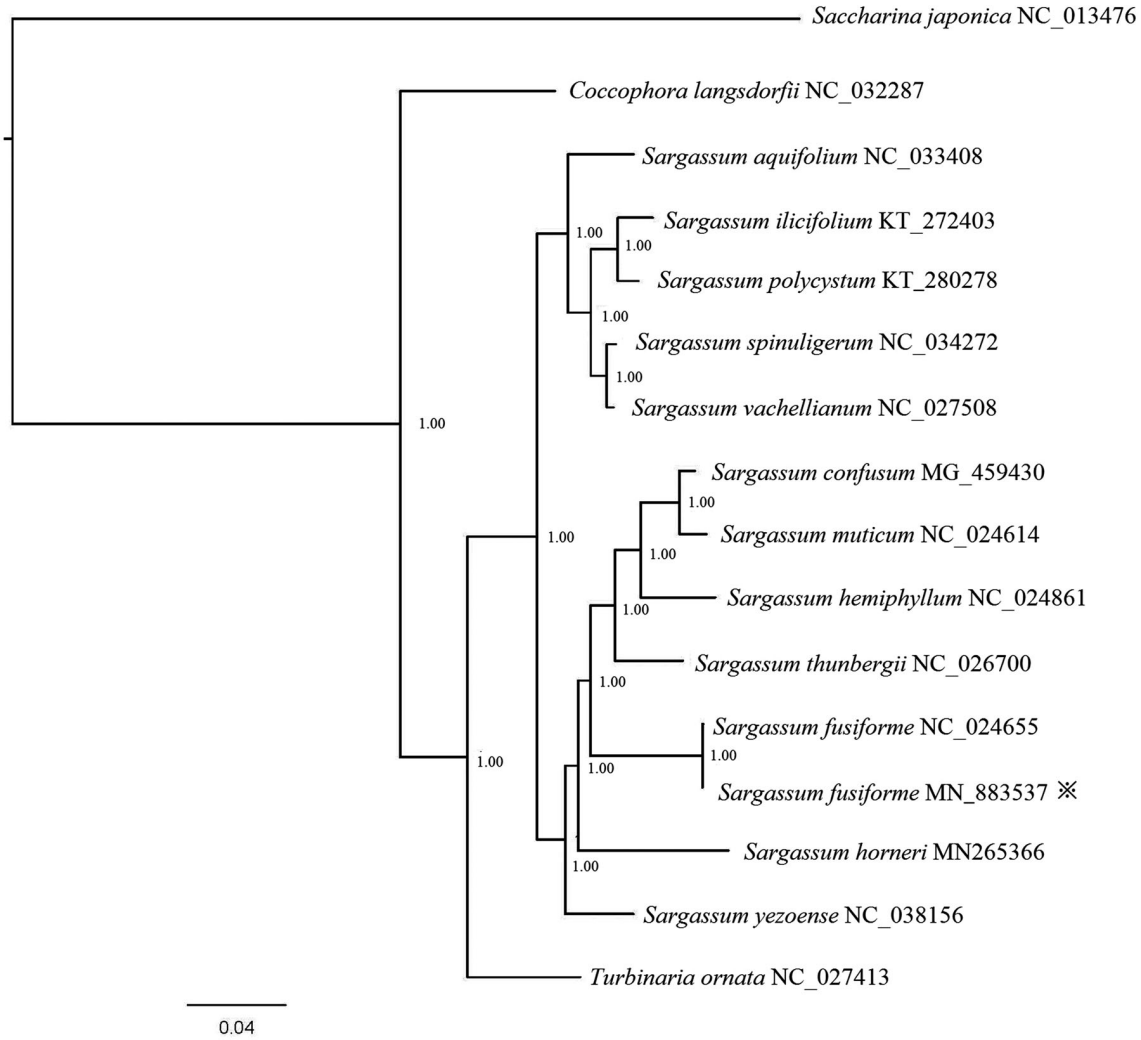
Phylogenetic tree based on all available complete mitochondrial genome of Sargassaceae family in the NCBI. Support values for each node were calculated from Bayesian posterior probability (BPP). Asterisks following species names indicate newly determined mitochondrial genomes.
